# *Neisseria meningitidis* carriage and risk factors among teenagers in Suizhou city in China

**DOI:** 10.1017/S0950268820002113

**Published:** 2020-09-14

**Authors:** Fei He, Hong mei Yang, Guo ming Li, Bing qing Zhu, Yating Zhang, Hong lin Jiang, Min Yuan, Yongzhong Jiang, Jing Lv

**Affiliations:** 1 Hubei Provincial Center for Disease Control and Prevention, Wuhan, PR China; 2 Chinese Center For Disease Control and Prevention, Beijing, PR China

**Keywords:** Carriage, *Neisseria meningitidis*, risk factors, teenagers

## Abstract

Teenagers are important carriers of *Neisseria meningitidis*, which is a leading cause of invasive meningococcal disease. In China, the carriage rate and risk factors among teenagers are unclear. The present study presents a retrospective analysis of epidemiological data for *N. meningitidis* carriage from 2013 to 2017 in Suizhou city, China. The carriage rates were 3.26%, 2.22%, 3.33%, 3.53% and 9.88% for 2013, 2014, 2015, 2016 and 2017, respectively. From 2014 to 2017, the carriage rate in the 15- to 19-year-old age group (teenagers) was the highest and significantly higher than that in remain age groups. Subsequently, a larger scale survey (December 2017) for carriage rate and relative risk factors (population density, time spent in the classroom, gender and antibiotics use) were investigated on the teenagers (15- to 19-year-old age) at the same school. The carriage rate was still high at 33.48% (223/663) and varied greatly from 6.56% to 52.94% in a different class. Population density of the classroom was found to be a significant risk factor for carriage, and 1.4 persons/m^2^ is recommended as the maximum classroom density. Further, higher male gender ratio and more time spent in the classroom were also significantly associated with higher carriage. Finally, antibiotic use was associated with a significantly lower carriage rate. All the results imply that attention should be paid to the teenagers and various measures can be taken to reduce the *N. meningitidis* carriage, to prevent and control the outbreak of IMD.

## Introduction

*Neisseria meningitidis* is one of the leading causative pathogens of invasive meningococcal disease (IMD), which remains a serious public health concern worldwide on account of its high morbidity and mortality, especially in children [1, 2]. *Neisseria meningitidis* is an obligate parasite in humans that tends to colonise the nasopharyngeal mucosa asymptomatically, and this is considered as healthy carriage of the parasite [3, 4].

Up to now, many studies all over the world have investigated the asymptomatic carriage of *N. meningitidis*. Currently, the carriage rate in healthy populations is highly variable, and ranges from 0% to 30% worldwide [5–19]. With regard to carriage according to age, teenagers have been found to have the highest carriage rate [5, 7, 10]. In fact, teenagers seem to be a potential reservoir of *N. meningitidis*; thus, this age group is most likely to be responsible for the transmission of *N. meningitidis* among the healthy population [11, 12]. Hence, in some countries, such as the USA and Britain, vaccination in teenagers is a common practice for reducing the carriage rate or preventing and controlling the outbreak of IMD in the healthy population [19, 20].

In the context of China, *N. meningitidis* surveillance has been carried out in many provinces and cities for several years. According to the reports, the carriage rate of asymptomatic carriers ranges from 1.03% to 9.88% [17, 21–24]. However, the asymptomatic carriage rate among teenagers is unclear, while they have the second highest incidence among different age groups. Therefore, it is necessary to monitor the carriage rate of *N. meningitidis* in teenagers in China, in order to assess and improve the present immunisation strategy.

For teenagers in China, there is no available immunisation strategy at present. Therefore, some fungible measures should be taken to control and prevent the IMD, including identifying and controlling the risk factors. Different risk factors have been reported, such as age, season, direct contact with a carrier or infected individual, overcrowding, socioeconomic status, passive smoking, immunologic status, pub patronage, partying, and so on [9, 12]. It is important to clearly identify these factors, so that at-risk individuals can be immunised and the transmission of this pathogen can be prevented.

A long-term *N. meningitidis* surveillance was conducted in Suizhou city, China. In this study, the surveillance data of the all age groups from 2013 to 2017 were systematically and retrospectively analysed. Subsequently, in December 2017, a larger scale survey was conducted among teenagers. As part of this survey, the participants were required to answer a short questionnaire that was designed to explore the risk factors for *N. meningitidis* carriage among teenagers. This is the first study to explore the risk factors associated with *N. meningitidis* carriage in a healthy population of teenagers in China, and the findings are important for designing an optimal immunisation and prevention strategy against IMD.

## Methods

### Retrospective analysis of epidemiological data from 2013 to 2017

Every year (from 2013 to 2017) during the epidemic season (usually in November to March of the following year), a healthy population was recruited on a voluntary basis in Suizhou city, China. The participants were distributed into seven age groups: <1, 1–2, 3–4, 5–6, 7–14, 15–19 and >20 years. There were at least 30 volunteers in each group. Oropharyngeal swab samples were collected (one sample from each volunteer). Pathogens were isolated from the collected samples.

The surveillance data of 2013–2017, including volunteers' information and isolation results, were collected and analysed. In 2017, the surveillance was conducted in November, all the volunteers of 15- to 19-year-old age group were recruited from three classes of one high school. All the students in this school lived in dormitories. The surveillance data from 2013 to 2017 were analysed retrospectively.

### A larger scale survey of *N. meningitidis* at the same school

Due to the carriage rate was noticeably high in the previous surveillance in November 2017, more volunteers of 15- to 19-year-old age group from the same school were recruited to perform a larger scale survey in December 2017. The volunteers were from 10 classes that were located in the same building in the school. They completed a short questionnaire that was designed to assess the risk factors associated with the carriage. The following information was included in the questionnaire: age, gender, immunisation history and antibiotics being taken in the last 30 days. The information of the classroom area and the time students spent in the classroom was collected from the school authority.

### Isolation and identification of *N. meningitidis*

One nasopharynx swab sample was collected from each volunteer with a sterile swab (3 M, USA). The sample was directly plated onto a selective chocolate agar plates (Dijing, Guangzhou city, Guangdong province, China), transported to the laboratory within 4 h, and incubated in a 5% (v/v) CO_2_ atmosphere at 37 °C for 24–48 h. The *N. meningitidis*-like colonies (round, smooth, moist, glistening, convex and grey) were sub-cultured on 5% sheep blood agar. *Neisseria meningitidis* was confirmed by Gram staining and standard biochemical tests using API NH (bioMérieux, Lyons, France). Genomic DNA was extracted using the Wizard Genomic DNA Purification Kit (Promega, USA) according to the manufacturer's instructions. PCR or real-time PCR was used to identify the genogroup of confirmed *N. meningitidis* isolates as described previously [25–27].

### Statistical analysis

All data were recorded using Microsoft Excel 2016 and double checked by an epidemiologist. After validation, the data were imported into SPSS 17.0 software for data analysis statistically. The carriage rates were calculated with 95% confidence interval (CI), and compared using the *χ*^2^ test. A *P* value of <0.05 was considered to indicate statistical significance. The multinomial logistic regression was conducted to analyse the risk factors affecting the carriage rate.

## Results

### Carriage patterns of *N. meningitidis* from 2013 to 2017

The carriage rate for 2013, 2014, 2015, 2016 and 2017 was 3.26% (CI 1.0–5.1%), 2.22% (CI 0.8–3.7%), 3.33% (CI 1.4–5.3%), 3.53% (CI 1.8–5.2%) and 9.88% (CI 7.5–12.3%), respectively (Table 1). The carriage rate in 2017 was significantly higher than that in 2013, 2014, 2015 and 2016 (*P* < 0.01 for all differences). In 2013, the carriage rate in the 3–4 age group is the highest. From 2014 to 2017, the carriage rate in the 15- to 19-year-old age group was the highest and significantly higher than that in remain age groups.

**Table 1.** Carriage rate of *N. meningitidis* in Suizhou city from 2013 to 2017

Age group (year)	2013		2014		2015		2016		2017^a^		2017^b^	
	Percentage (*n*/*N*)^c^	95% CI	Percentage (*n*/*N*)^c^	95% CI	Percentage (*n*/*N*)^c^	95% CI	Percentage (*n*/*N*)^c^	95% CI	Percentage (*n*/*N*)^c^	95% CI	Percentage (*n*/*N*)^c^	95% CI
<1	3.70% (1/27)	−3.9 to 11.3%	0 (0/38)	NA	0 (0/40)	NA	0 (0/87)	NA	0 (0/30)	NA		
1–2	3.45% (1/29)	−3.6 to 10.5%	0 (0/23)	NA	3.33% (1/30)	−3.5 to 10.2%	0 (0/81)	NA	0 (0/31)	NA		
3–4	8.93% (5/56)	1.2–16.7%	1.89% (1/53)	−1.9 to 5.7%	0 (0/43)	NA	0 (0/82)	NA	0 (0/99)	NA		
5–6	5.00% (3/60)	−0.7 to 10.7%	0 (0/48)	NA	0 (0/43)	NA	0 (0/80)	NA	0 (0/94)	NA		
7–14	0.92% (1/109)	−0.9 to 2.7%	2.21% (3/136)	−0.3 to 4.7%	1.89% (1/53)	−1.9 to 5.7%	6.67% (2/30)	−2.8 to 16.1%	1.16% (1/86)	−1.1 to 3.5%		
15–19	2.94% (1/34)	3.0–8.9%	7.69% (4/52)	0.2–15.2%	13.04% (6/46)	2.9–23.2%	46.67% (14/30)	27.7–65.6%	28.36% (57/201)	22.1–34.6%	33.63%(223/663)	30.0–37.2%
>20	0 (0/53)	NA	1.79% (1/56)	−1.8 to 5.4%	4.00% (3/75)	−0.5 to 8.5%	0 (0/63)	NA	0 (0/46)	NA		
Total	3.26% (12/368)	1.0–5.1%	2.22% (9/406)	0.8–3.7%	3.33% (11/330)	1.4–5.3%	3.53% (16/453)	1.8–5.2%	9.88% (58/587)	7.5–12.3%	33.63%(223/663)	30.0–37.2%

CI, confidence interval.

a The survey was carried out in November 2017.

b The survey was carried out in December 2017.

^c^*n* = the number of *N. meningitidis* strains, *N* = the number of samples.

From 2013 to 2017, a total of 106 *N. meningitidis* were isolated, of which 57 were genogrouped as B (*n* = 53), C (*n* = 3) and W (*n* = 1), while the other 49 *N. meningitidis* strains cannot be genogrouped (Table 2).

**Table 2.** Genogroup of *N. meningitidis* strains from 2013 to 2017

Year	Samples (*N* = 2807)	*B* (*n* = 114)	*C* (*n* = 13)	*W* (*n* = 1)	Non-genogroup (*n* = 201)	Total no. of isolates (*n* = 329)
2013	368	2	0	0	10	12
2014	406	5	0	0	4	9
2015	330	7	0	0	4	11
2016	453	11	1	1	3	16
2017^a^	587	28	2	0	28	58
2017^b^	663	61	10	0	152	223

*n* means the number of *N. meningitidis* strains, *N* means the number of samples.

a The survey was carried out in November 2017.

b The survey was carried out in December 2017.

### Carriage patterns of *N. meningitidis* for the 15- to 19-year-old group in 2017

**Fig. 1.**
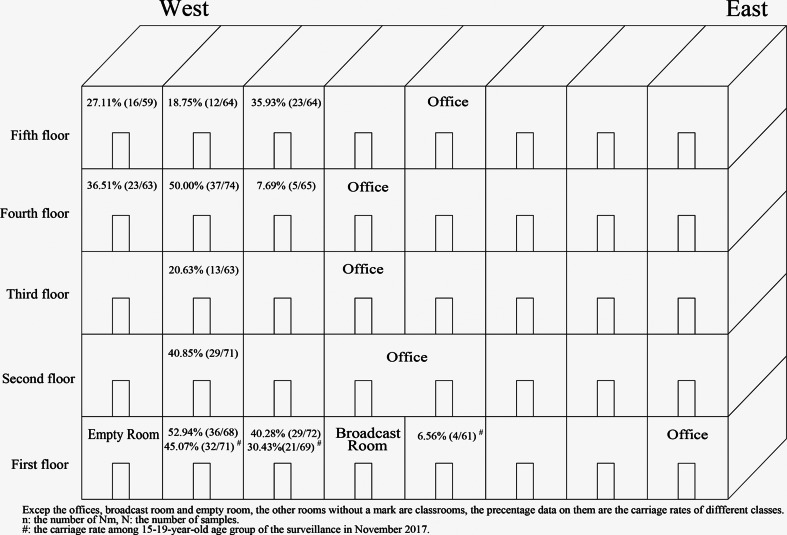
Carriage rates for different classes according to location. The carriage rates (data in every classroom) varied greatly in different classes. In the two classes recruited both in November 2017 and December 2017, there was no significant difference in carriage rate between the two surveys.

In November 2017, a total of 201 volunteers from the 15- to 19-year-old group were recruited from three classes (61, 71 and 69 volunteers from each class) in a high school. A total of 57 strains were isolated from the 201 samples. The carriage rates for the three classes were 6.56% (4/61), 45.07% (32/71) and 30.43% (21/69) (*χ*^2^ = 24.177, *P* < 0.001), even though the three classes were located in the same building (Fig. 1).

In December 2017, a larger scale survey was conducted in the same age group (15−19 years), and it included 663 volunteers from 10 classes (including volunteers from two of the three classes in the November 2017 surveillance) in the same building of the same school. The 10 recruited classes were distributed from the first floor to the fifth floor (Fig. 1). The total carriage rate from this survey was calculated as 33.63% (223/663, CI 30.0–37.2%) (Table 1) and the carriage rates for the 10 classes ranged from 7.69% to 52.94% (Table 3). Interestingly, the carriage rates varied greatly even among the classes on the same floor (Fig. 1). For the two classes that were recruited in both surveys in 2017, there was no significant difference in carriage rate between the two surveys (45.07% (32/71) *vs.* 52.94% (36/68), *χ*^2^ = 0.861, *P* = 0.353; 30.43% (21/69) *vs.* 40.28% (29/72), *χ*^2^ = 1.492, *P* = 0.222).

**Table 3.** Correlation of carriage rate with population density in 2017 (%, *n*/*N*)^a^

Classes	Population density in the classroom (persons/m^2^)	Carriage rate			Correlation co-efficient between males and females	
		Different classes, 95% CI	Males	Females	*χ*^2^ and *P* value	OR, 95% CI
2 (11)	1.30 (65/50)	7.69% (5/65), 1.0–14.3%	8.33% (1/12)	7.55% (4/53)	*P* = 1.00^b^	0.898, 0.091–8.893
2 (3)	1.28 (64/50)	18.75% (12/64), 8.9–28.6%	27.78% (10/36)	7.14% (2/28)	*χ*^2^ = 4.402, *P* = 0.036	0.200, 0.040–1.003
2 (4)	1.26 (63/50)	20.63% (13/63), 10.4–30.9%	28.95% (11/38)	8.00% (2/25)	*χ*^2^ = 4.040, *P* = 0.044	0.213, 0.043–1.064
2 (1)	1.18 (59/50)	27.11% (16/59), 15.4–38.8%	15.15% (5/33)	42.31% (11/26)	*χ*^2^ = 5.426, *P* = 0.020	4.107, 1.201–14.038
2 (5)	1.28 (64/50)	35.93% (23/64), 23.9–48.0%	45.45% (10/22)	30.95% (13/42)	*χ*^2^ = 1.319, *P* = 0.251	0.538, 0.186–1.559
2 (7)	1.26 (63/50)	36.51% (23/63), 24.3–48.7%	43.59% (17/39)	25.00% (6/24)	*χ*^2^ = 2.215, *P* = 0.137	0.431, 0.141–1.322
2 (14)	1.44 (72/50)	40.28% (29/72), 28.7–51.9%	50.00% (17/34)	31.58% (12/38)	*χ*^2^ = 2.531, *P* = 0.112	0.462, 0.177–1.204
2 (10)	1.42 (71/50)	40.85% (29/71), 29.1–52.6%	42.11% (16/38)	39.39% (13/33)	*χ*^2^ = 0.054, *P* = 0.817	0.894, 0.346–2.311
2 (9)	1.48 (74/50)	50.00% (37/74), 38.3–61.7%	51.92% (27/52)	45.45% (10/22)	*χ*^2^ = 0.259, *P* = 0.611	0.772, 0.284–2.098
2 (13)	1.42 (71/50)	52.94% (36/68), 40.8–65.1%	68.75% (22/32)	38.89% (14/36)	*χ*^2^ = 6.063, *P* = 0.014	0.289, 0.106–0.789
Total	1.33 (666/500)	33.63% (223/663), 30.0–37.2%	40.48% (136/336)	26.61% (87/327)	*χ*^2^ = 14.284, *P* < 0.001	1.876, 1.351–2.604

OR, odds ratio; CI, confidence interval.

a *n* = the number of *N. meningitidis* strains, *N* = the number of samples.

^b^When *χ*^2^ test, the except count of some data was less than 5, and the *χ*^2^ cannot be obtained.

During the larger scale survey for the volunteers in the 15–19-year age group, a total of 223 *N.meningitidis* were isolated, of which 71 were genogrouped as B (*n* = 61) and C (*n* = 10), while the other 152 *N. meningitidis* strains cannot be genogrouped (Table 2).

### Risk factors for *N. meningitidis* carriage in the 15- to 19-year-old group

The available risk factors for *N. meningitidis* carriage, including vaccination status, population density of the classroom, time spent in the classroom, gender and antibiotic use in the last 30 days, were analysed. In December 2017, all the volunteers had not been vaccinated with the meningococcal polysaccharide vaccine/conjugate vaccine, so it was difficult to determine whether vaccination was a risk factor.

**Fig. 2.**
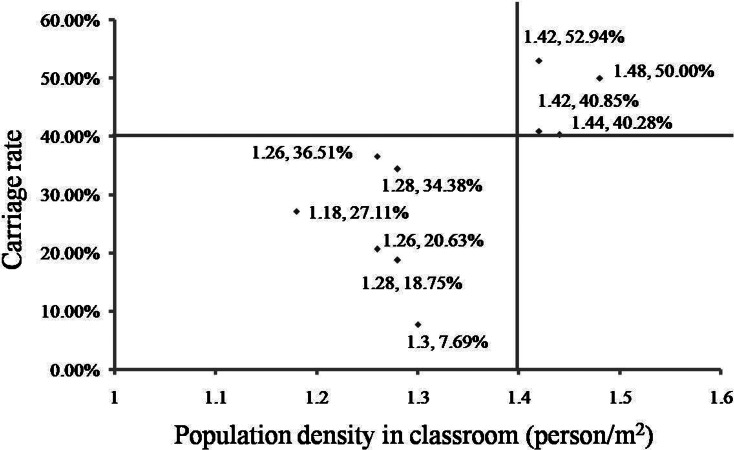
Association between population density and carriage rate. The carriage rate and the population density for all the recruited class are shown in the figure. A density of 1.4 persons/m^2^ might be a critical point beyond which the carriage rates increase to above 40%. The carriage rate for classes with a population density of over 1.4 persons/m^2^ is significantly higher than that for classes with a density below 1.4 persons/m^2^.

All the classrooms included in this study had an area of 50 m^2^. The population density of each classroom was calculated by dividing the number of students by the classroom area (Table 3), a density of 1.4 persons/m^2^ might be a critical point beyond which the carriage rates increase to above 40% (Fig. 2). There are six classes with a population density of below 1.4 persons/m^2^, from which a total of 378 samples were obtained and the carriage rate was 24.34% (92/378). While the other four classes have a population density of over 1.4 persons/m^2^, from which a total of 285 samples were obtained and the carriage rate was 45.96% (131/285). The carriage rate for classes with a population density of over 1.4 persons/m^2^ is significantly higher than that for classes with a density below 1.4 persons/m^2^ (*χ*^2^=34.045, *P* < 0.001, OR 2.644, CI 1.900–3.681).

Among the 10 surveyed classes, students in the class with the lowest carriage rate (7.69%, 5/65) only spent 70% of the time (10 h) than the students in other nine classes (36.45%, 218/598) spent (14 h) (*χ*^2^=21.728, *P* < 0.001, OR 6.884, CI 2.723–17.403). Therefore, time spent in the classroom is an important risk factor associated with the carriage.

The carriage rate of male students was significantly higher than of female students (40.48% (136/336) *vs.* 26.61% (87/327), *χ*^2^=14.284, *P* < 0.001, OR 1.876, CI 1.351–2.604). The carriage rate in male students (15.15%, 5/33) was significantly lower than that in female students (42.31%, 11/26) in only one of the 10 classes (*χ*^2^=5.426, *P* = 0.020). In the other nine classes, the carriage rate was always higher in the male students, but the difference was significant in only three classes (*P* < 0.05 for all three classes) (Table 3).

There was also a significant difference in carriage rates between volunteers who had taken antibiotics (17.91%, 12/67) and those who had not taken antibiotics, and antibiotic taken (35.40%, 211/596) was associated with a significantly lower carriage rate (*χ*^2^ = 8.256, *P* = 0.004, OR 2.512, CI 1.316–4.796).

In bivariable logistic regression analysis, associated factors with a *P* value <0.2 in univariate were transferred to multivariable logistic regression to the significant association of these factors. Multivariable logistic statistical analysis showed that the factors statistically significantly associated with the risk of being meningococcal carriage were: gender (OR 1.688; CI 1.200–2.376; *P* = 0.003), population density of the classroom (OR 2.119; CI 1.497–3.000; *P* = 0.000) and time spent in the classroom (OR 3.699; CI 1.422–9.626; *P* = 0.007). While antibiotic taken in the last 30 days (OR 1.684; CI 0.857–3.306; *P* = 0.130) were not associated with the carriage status (Table 4).

**Table 4.** Factors associated with meningococcal carriage

Variables	Values	Positive (*n* = 223)	Negative (*n* = 440)	Univariate analysis		Multivariate analysis^a^	
		*N* (%)	*N* (%)	OR (95% CI)	*P* value	OR (95% CI)	*P* value
Population density of the classroom	Below 1.4 persons/m^2^	92 (41.26)	286 (65.00)	REF	*P* = 0.000	REF	*P* = 0.000
	Over 1.4 persons/m^2^	131 (58.74)	154 (35.00)	2.644 (1.900–3.681)		2.119 (1.497–3.000)	
Time spent in the classroom	10 h	5 (2.24)	60 (13.64)	REF	*P* = 0.000	REF	*P* = 0.007
	14 h	218 (97.76)	380 (86.36)	6.884 (2.723–17.403)		3.699 (1.422–9.626)	
Gender	Female	87 (39.01)	240 (54.55)	REF	*P* = 0.000	REF	*P* = 0.003
	Male	136 (60.99)	200 (45.45)	1.927 (1.389–2.680)		1.688 (1.200–2.376)	
Antibiotic taken in the last 30 days	Yes	12 (5.38)	55 (12.50)	REF	*P* = 0.005	REF	*P* = 0.130
	No	211 (94.62)	385 (87.50)	0.398 (0.209–0.760)		1.684 (0.857–3.306)	

OR, odds ratio; CI, confidence interval; REF, reference value.

Univariate and multivariate analyses. Suizhou, China (*n* = 663).

a Multivariate regression model including variables with *P* < 0.2 at the univariate analysis.

## Discussion

In this study, the carriage patterns of *N. meningitidis* in Suizhou city of China were investigated during the last few years (2013–2017). The findings showed that the carriage rate was the highest in teenagers (15–19-year-old age group) from 2014 to 2017, and increased by years. The carriage rate had increased up to 28.36% in 2017. However, the carriage rates in the other age group did not increase obviously, or decrease to some degree. In accordance with the present findings, other studies have also shown that teenagers are a potential reservoir of *N. meningitidis*, and they are most likely to be responsible for the transmission of *N. meningitides* in the healthy population [5, 7, 10–12]. Hence, much more attention should be paid to teenagers in China.

In some countries, vaccination is provided for teenagers to reduce the carriage rate among the teenagers and the incidence of IMD among the whole population. The available vaccines differ across countries and include both conjugate and polysaccharide vaccines, but the use of only conjugate vaccines could interrupt the acquisition of carriage [8, 28]. In the USA, vaccines are not only provided to high-risk populations, but also designed for different age groups, for example, the conjugate vaccine MenACWY is recommended for 11- to 21-year-olds, and MenB is recommended for 16- to 23-year-olds [29]. Furthermore, from 2007, MenACWY has been recommended for all adolescents, and adolescents may also be immunised as a stand-alone strategy [19, 29–31]. In 2013, in the UK, one dose of vaccine that was earlier recommended for infants was recommended for adolescents of age 14–15 years for reducing the carriage rate and interrupting the transmission of carriage [20, 31]. However, in most countries, *N. meningitidis* vaccines are generally not included in the expanded programme on immunisation and provided to high-risk groups only. In China, only polysaccharide vaccines are included in the expanded programme on immunisation for the high-risk population (infants and children). According to the increasing carriage rate in the 15- to 19-year-old age group, it is important for China to consider following the example of other countries and modifying the immunisation strategy to use conjugate vaccine against IMD-related serogroups in teenagers.

On the contrary, there are many risk factors in the foreign countries associated with the acquisition and carriage of *N. meningitidis* for teenagers, such as living in dormitories, sharing utensils or beverages, frequenting bars, passive smoking, often partying and so on [11, 12]. Compared with the foreign students, the teenager volunteers are not allowed to attend the bars, smoke passively and attend parties usually, and also all of them lived in dormitories. Hence, the above risk factors related to the teenagers are not to be included. Instead, the other relevant risk factors such as vaccination status, population density in the classroom, time spent in the classroom, gender and antibiotic use in the last 30 days were analysed. Since there are no studies that have reported the relationship between the carriage rate and population density and time spent in the classroom, these findings are highly significant.

In the present study, the amount of time spent in the classroom emerged as another important risk factor associated with the carriage. The less time spent in the classroom, the lower was the carriage rate. As it is difficult for teenagers to reduce the amount of time spent in the classroom, it is recommended that they try to increase the amount of time spent outdoors. Regrettably, it is difficult to shed light on the optimal ratio of time spent in the classroom and time spent outdoors. Therefore, we propose that teachers try to give students as much time as possible outdoors.

Previous studies have shown that males were more likely carriers than females [32–35]. In the present study, the carriage rate, overall, was also significantly higher in the male students than in the female students. This risk factor needs to be further examined, as the present findings are specific to teenagers only and might be different for the population as a whole. In particular, more comprehensive data from Suizhou are required to understand better this gender-based effect.

The carriage is the result of interaction between the asymptomatic carriers and pathogen. In the present study, some interesting findings of the carriage pattern and risk factors for the teenagers were identified. These results are from the perspective of the host. The researches of the molecular typing, clonal complex, serotyping, adhesion, antibiotic resistance and so on were conducted for the strains, in order to understand the carriage pattern better and improve the strategy of IMD prevention and control. Therefore, further research should be carried out on the isolates obtained in order to understand the carriage and transmission of this pathogen and evaluate the present immunisation strategy in China.

## Data Availability

The findings in this study do not rely on any data, code or other resource.
